# Built environment and physical activity: domain- and activity-specific associations among Brazilian adolescents

**DOI:** 10.1186/s12889-017-4538-7

**Published:** 2017-07-03

**Authors:** Inácio Crochemore Mohnsam da Silva, Adriano Akira Hino, Adalberto Lopes, Ulf Ekelund, Soren Brage, Helen Gonçalves, Ana B Menezes, Rodrigo Siqueira Reis, Pedro Curi Hallal

**Affiliations:** 1 Post-graduate Program in Epidemiology of Federal University of Pelotas, Rua Marechal Deodoro 1160, Pelotas, RS 96020-220 Brazil; 2 Physical Activity and Quality of Life Research Group, Pontifical Catholic University of Paraná, Curitiba, Brazil; 3 Medical Research Council Epidemiology Unit, University of Cambridge, Cambridge, UK; 4 Department of Sport Medicine, Norwegian School of Sport Sciences, Oslo, Norway; 5 Prevention Research Center, Brown School, Washington University in St. Louis, St. Louis, USA; 6 Post-Graduate Program in Physical Education, Federal University of Parana, Curitiba, Brazil; 7 Health Technology Graduate Program - PPGTS, Polytechnic School, Pontifical Catholic University of Paraná, Curitiba, Brazil

**Keywords:** Environment, Public open spaces, Leisure-time, Commuting physical activity

## Abstract

**Background:**

Physical activity is likely to be determined as a complex interplay between personal, interpersonal, and environmental factors. Studying the built environment involves expanding the focus from the individual perspective to a public health one. Therefore, the objetive of this study was to examine the association between the built environment and objectively-measured physical activity among youth.

**Methods:**

Cross-sectional analysis of data from of a Brazilian birth cohort during adolescence. Physical activity was measured using accelerometers (GENEActiv) and self-report (International Physical Activity Questionnaire, long version). Participants’ home addresses were geocoded and built environment characteristics such as streets’ pattern and quality, and public open spaces attributes for physical activity practice were evaluated in a 500-m circular buffer surrounding their homes.

**Results:**

A total of 3379 participants were included. Street lighting (β = 2.2; 95%CI: 0.5; 3.9) was positively associated with objectively-measured moderate-vigorous physical activity (MVPA) and proportion of paved streets and buffer’s average family income were associated with lower MVPA. Living near the beach increased the odds of leisure-time MVPA practice by 3.3 (95%CI: 1.37; 8.02) times. There was a built environment-by-socioeconomic status (SES) interaction for the associations with commuting physical activity; street lighting [Odds ratio (OR) = 1.22; 95%CI: 1.01; 1.47] and presence of cycle lanes (OR = 1.77; 95%CI: 1.05; 2.96) were positively associated with commuting physical activity only among the intermediate SES tertile.

**Conclusion:**

Beachfront, street lighting, paved streets and cycle lanes were associated with physical activity patterns. This suggests that infrastructure interventions may influence physical activity levels of Brazilian adolescents.

## Background

Globally, four out of five adolescents do not achieve the recommended 60 min per day of moderate-to-vigorous intensity physical activity [1]. Every year, physical inactivity causes 5.3 million deaths [2]. Adolescence (and early adulthood) has been suggested as a critical period for physical activity interventions, due to the substantial decline in physical activity between this age and early adulthood [3, 4]. Moreover, active youth tend to be more active in adulthood [5].

Physical activity is likely to be determined as a complex interplay between personal, interpersonal, and environmental factors, operating differentially in the main activity domains. The ecological model adapted for this behaviour assumes physical activity as a consequence of different factors organized in multiple levels of influence (individual, interpersonal, environmental, policy and global) [3]. Furthermore, a complex system approach highlights the non-linearity of these multiple levels of influence and reinforces the existence of many others factors which might act as enablers, accelerants, or synergies of multiple influences [6].

Research on the association between environmental factors and youth physical activity has increased in recent years. Studying this association involves expanding the focus from the individual perspective to a whole-societal perspective, in which people and places influence behaviours such as physical activity [7]. Built environment is the physical form of communities [8], including among other characteristics, design and network of streets, sidewalks, bicycle lanes, green space, public and private recreation facilities. Built environment characteristics can be assessed subjectively, according to the participant’s perception, and objectively, by either direct observation or through Geographic Information System (GIS) [8, 9], the latter provides a framework to use available information that has some spatial reference.

A systematic review stated that neighbourhood design, recreation facilities, and transportation systems seem to be the most consistently characteristics associated with physical activity among youth, and that more evidence on the association between youth physical activity and the built environment in low- and middle-income countries is needed [3]. Therefore, exploratory-based analyses are still relevant in scenarios where there is not enough evidence of either specific or combined environmental features determining physical activity. The aim of the present study was, therefore, to examine the association between the built environment characteristics and physical activity among youth (18y) from the 1993 Pelotas (Brazil) birth cohort, as well as to evaluate potential sex and socioeconomic interactions in the association of interest.

## Methods

The current study is based on cross-sectional analyses from the most recent follow-up visit of a birth cohort started in 1993 in Pelotas (Brazil). The city is markedly flat and is located in the extreme south of Brazil, with around 320,000 inhabitants. All children born in hospitals of mothers living in the city of Pelotas in the calendar year of 1993 were eligible to participate of the cohort study. There were 5265 births (refusals accounted for less than 1%) and 5249 took part [10]. Participants have been followed up periodically and the last follow-up was carried out in 2011/2012 when participants were aged 18.4 (SD 0.3) years [11].

Cohort members were invited to visit the research clinic at the Epidemiological Research Center of the Federal University of Pelotas, where the follow-up took place through clinical exams, psychological tests and questionnaire administration. Participants were also invited to wear an accelerometer following the clinical visit. Details of the protocol are available elsewhere [4, 12]. The cohort study was approved by the School of Medicine Ethics Committee of the Federal University of Pelotas (protocol number: 05/11). All participants voluntarily signed a consent letter prior to participating in the study.

Total physical activity was objectively evaluated by accelerometry and leisure- and commuting physical activity were assessed by self-report. Participants worn a triaxial raw-data accelerometer (GENEActiv; ActivInsights, Kimbolton, UK) attached to their non-dominant wrist, reporting acceleration in m*g* (gravitational equivalent, 1000 m*g* = 1 *g*). Free-living physical activity was assessed in a 24 h–protocol for a period from 4 to 7 days. Non-wear time was defined based on the standard deviation and value range of each accelerometer axis in 60-min windows with 15-min moving increments. A time window was considered as non-wear time when, for at least two out of the three axes, the standard deviation was lower than 13 m*g* and the value range was lower than 50 mg. [13] We calibrated the sensors to local gravity [14] and analysed the raw data in 5 s epochs, from which we derived average minutes per day spent in moderate to vigorous physical activity (MVPA) calculated as time when acceleration was above 100 m*g* in 10 min bouts. [4] Participants with at least 2 days of measurement (a valid day must have at least 16 h), at least one complete cycle of 24 h and calibration errors lower than 0.02 *g*. Further details of the accelerometry-based data collection and procedures of analyses are available elsewhere [4, 12–14].

Self-reported leisure and commuting physical activities were assessed by their respective sections of the long form of the International Physical Activity Questionnaire (IPAQ) [15]. The recall period was the last 7 days prior to the interview and only activities lasting more than 10 consecutives minutes were considered. In terms of leisure time, activity-specific estimates were only performed separating participants between those walking or not, and those practicing moderate to vigorous activities during leisure time or not. Commuting physical activity comprised bicycle and walking for any purpose (e.g. to and from work or school) practiced in the previous week, which then classified subjects as active or passive commuters.

Built environment indicators were assessed objectively. Participants’ geocode and environmental variables were created using GIS through the Esri-ArcGIS software. Cohort members were geocoded based on Pelotas’ streets network, provided by the Mobility and Management City Secretary. The street network was updated in 2008 and presents information about the initial and final number of each street block. Thus, participants’ geocoding was carried out linking their home address. Geographic coordinates were also collected in Google maps for around 15% of the participants, as a complementary strategy for geocoding. Participants who were living in rural areas of Pelotas or other cities were not eligible for geocoding due the unavailability of environmental characteristics to be analysed (*N* = 397, 9.7% of those followed up).

A circular buffer of 500 m (m) radius surrounding each participant’s home was used to assess the built environment variables. The buffer was defined arbitrarily considering that it would include all possible destinations reached by 10-min brisk walk. Population density (overall and population between 16 and 20 years old), average area income and proportion of street lighting, paved streets, sidewalks, trees existence, street garbage and open sewage for each individual buffer were assessed based on National Demographic Census performed in 2010 [16]. Census’ interviewers were trained to evaluate the previously mentioned environmental characteristics in front of participants’ households, and therefore, the proportion of households presenting such characteristics in each census tract are available. However, as these variables were based on census tracts, each individual buffer environment was determined through the average values of all census tracts intersected by them.

Street connectivity was assessed as the number of 4-way intersections in each buffer according to the street network provided by Mobility and Management City Secretary of Pelotas. A sub-study was carried out in-person by a trained team to identify which green areas were referred to as public open spaces, their quality and suitability to physical activity practice. From 700 green areas, only 245 were considered public open spaces and were evaluated using the Physical Activity Resource Assessment (PARA) [17]. PARA allows a comprehensive assessment of a variety of features, including both quantity and quality for physical activity practice (e.g. sports courts, walking paths, public gyms, among others). However, the current analyses are limited to: (a) number of public open spaces, (b) number of public open spaces with at least one physical activity attribute, (c) number of public open spaces with some physical activity attribute of at least ‘regular’ quality and (d) number of public open spaces with some physical activity attribute of at least ‘good’ quality. We marked an attribute as having good quality when it is present, and its structure was completely available and able to be readily used. We classified an attribute as having regular quality when it was not in ideal conditions or presented some structure lacking. In addition, information on number of football pitches and walking areas and other physical activity attributes for each buffer was also estimated using the GIS information. We selected these two attributes because they are the most frequently observed in Pelotas, Brazil. A variable summing up all attributes supporting physical activity practice was also generated.

All cycle paths/lanes in the city of Pelotas were measured by a GPS device in August 2012 and included as a layer in the ArcGIS software. Beachfront was added based on an orthophotograph. Thus, variables of cycle paths/lanes number in each buffer and the beachfront existence were generated.

All gyms (private health clubs) were geocoded using a database built upon a previous survey conducted in 2012. This information allowed the identification of the number of gyms within each individual buffer. Finally, the closest distance between participant’s households to any place supporting physical activity practice was also determined. A summary description of each outcome and environmental exposure is presented in Table 1.

**Table 1:** Description of outcome and exposure variables (analytical sample, *N* = 3379)

Variable	Units	Mean (SD)	Median	Min-Max	N (%)
Outcome variables – physical activity					
Total MVPA – Minutes per day spent in moderate to vigorous physical activity (accelerometry)	Minutes/day	42.9 (42.7)	31.5	0–499	-
Leisure walking – Minutes per week spent in walking during leisure time	Minutes/week	59.2 (150.4)	0	0–2100	-
Any leisure walking (Yes/No)	Proportion	-	-	-	1089 (32.3)
Leisure MVPA – Minutes per week spent in moderate to vigorous physical activity during leisure time	Minutes/week	336.5 (520.4)	120.0	0–4680	-
Any leisure MVPA (Yes/No)	Proportion	-	-	-	2021 (60.0)
Commuting physical activity – Minutes per week spent in commuting physical activity	Minutes/week	273.7 (467.1)	150	0–9300	-
Any commuting physical activity (Yes/No)	Proportion	-	-	-	2953 (88.3)
Environmental variables					
INFORMATION BASED ON DEMOGRAPHIC CENSUS 2010 AND STREET NETWORK PATTERN					
Population density – Population density of the buffer	People/km^2^	6353 (2843)	6401	28–16,801	-
Population aged 16–20 y density – Population density of people aged 16–20 years of the buffer	People/km^2^	637 (310)	598	2–1775	-
Average family income per capita – Average family income per capita of the buffer	Minimum wage	0.7 (0.1)	0.7	0.4–1.1	-
Connectivity between four or more streets – Number of at least four –way intersections in the buffer	Number	18 (13)	16	0–79	-
Street lighting proportion – Proportion of households with near public street lighting in the buffer	Proportion	0.87 (0.19)	0.95	0–1	-
Paved streets proportion – Proportion of households located in paved streets, in the buffer	Proportion	0.17 (0.26)	0.01	0–0.95	-
Sidewalks proportion – Proportion of households with sidewalks in front, in the buffer	Proportion	0.25 (0.30)	0.1	0–1	-
Trees existence proportion – Proportion of households presenting trees around, in the buffer	Proportion	0.80 (0.20)	0.85	0–1	-
Open sewage proportion – Proportion of households with open sewage in front, in the buffer	Proportion	0.34 (0.25)	0.32	0–1	-
Spread garbage proportion – Households with spread garbage in front, in the buffer	Proportion	0.06 (0.06)	0.03	0–0.5	-
PHYISICAL ACTIVITY ATTRIBUTES					
Public open spaces – Number of public open spaces (parks and boulevards) in the buffer	Number/Proportion^a^	3.2 (3.0)	2	0–19	2870 (86.3)
Public open spaces with at least on physical activity attribute – Number of public open spaces with at least one physical activity attribute regardless its quality	Number/Proportion^a^	2.1 (2.1)	1	0–11	2526 (75.9)
Public open spaces with at least on physical activity attribute of ‘regular’ quality – Number of public open spaces with at least one physical activity attribute considered of regular quality	Number/Proportion^a^	1.6 (1.8)	1	0–11	2170 (65.2)
Public open spaces with at least on ‘good’ physical activity attribute – Number of public open spaces with at least one physical activity attribute considered of good quality	Number/Proportion^a^	0.8 (1.3)	0	0–9	1411 (42.4)
Walking paths/trails – Number of walking paths/trails in the buffer	Number/Proportion^a^	0.9 (1.8)	0	0–11	884 (26.6)
Football pitches – Number of football pitches in the buffer	Number/Proportion^a^	1.5 (2.1)	1	0–11	1959 (58.9)
Cycle paths/lanes – Number of cycle paths/lanes in the buffer	Number/Proportion^a^	0.3 (0.6)	0	0–3	806 (23.9)
Gym – Number of private gyms in the buffer	Number/Proportion^a^	2 (3.7)	1	0–35	2165 (64.1)
Beachfront – Existence of beachfront in the buffer	Proportion	-	-	-	42 (1.2)
Total attributes for physical activity practice – Number of physical activity attributes in the buffer (open sports places, walking and cycle paths/trails, beachfront and gym)	Number/Proportion^a^	5.3 (5.4)	4	0–20	2997 (90.1)
Lower distance for any physical activity attribute – Lower distance to arrive in any physical activity attribute (open sports places, walking and cycle paths/trails, beachfront and gym)	Meters	277 (295)	218	0–3900	-

*SD* standard deviation

*Min-Max* minimum and maximum values

^a^Proportion of existence of at least one attribute by buffer

Linear and Logistic regressions were performed in the crude and adjusted analyses. In the adjusted model the covariates were sex, time living in that address, and socioeconomic status (SES) generated by a standardized socioeconomic questionnaire, which included questions on household assets and educational level. We also performed additional adjustment for other environmental factors that were associated with the specific outcome under investigation, except when environmental variables presented high collinearity (rho ≥ 0.6). Interaction tests were conducted to test whether the association between the environment and physical activity varied across gender and socioeconomic groups.

The following variables were standardized (transformed into Z-scores) in order to provide results allowing the assessment of their relative importance: population density (overall and between 16 and 20 years), average family income per capita, proportion of street lighting, paved streets, sidewalks, trees existence, open sewage, spread garbage, number of connectivity between four or more streets, and closest distance to any physical activity facility. Associations were tested only when there was a plausible rationale in the literature considering environmental attributes and specific physical activity domains. Statistical analyses were conducted using Stata 12.1®. A significance level of 5% was used.

## Results

**Fig. 1:**
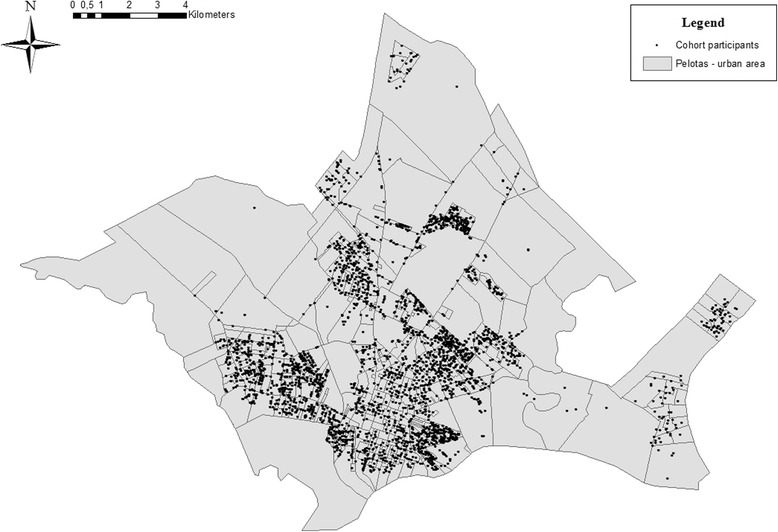
Pelotas (1993) birth cohort members geocoding (*N* = 3379). *Pelotas’ streets network was provided by the Mobility and Management City Secretary and the geocoding was performed using Esri-ArcGIS software

The follow-up rate was 81.3%. From 3709 participants living in urban Pelotas, 3379 were geocoded (Fig. 1). Issues with address information and incompatibilities in the street network were responsible for 330 missing data (8.1% of those eligible). Participants accumulated on average 42.9 (SD 42.7) minutes of MVPA in 10-min bout per day as measured by accelerometry. The proportion of participants reporting any leisure-time walking was 32.3% whereas; 60.0% reported any leisure-time MVPA, and 88.3% reported any commuting physical activity (Table 1).

According to census information attributed to each buffer, 87% of the households had public street lighting and 80% of the households had trees on the street. A low proportion of households had sidewalks (25%) and only 17% were located in paved streets. In each cohort member’s buffer, there were, on average, 2.1 public open spaces with at least one physical activity attribute. Moreover, at least one walking paths/trails was observed in 26.6% of the buffers (Table 1).

Crude and adjusted associations between built environment characteristics and objectively measured MVPA are presented in Table 2. Public open spaces and variables related to attributes of physical activity practice were inversely associated with MVPA only in the crude analyses. Only three environmental predictors were associated with MVPA in the final adjusted model out of the 21 examined; higher proportion of street lighting (β = 2.2; 95%CI: 0.5; 3.9, *p* = 0.013) was positively associated with objectively measured MVPA. In the opposite direction, higher proportion of paved streets and buffer’s average family income were associated with lower MVPA (Table 2).

**Table 2:** Crude and adjusted associations between built environmental variables and total moderate to vigorous physical activity (average daily minutes of 10-min bouts/day based on accelerometry) (*N* = 3379)

Variables	Crude analyses			Adjusted analyses**		
	β	95%CI	p^#^	β	95%CI	p^#^
Population density^¥^	1.6	0.1; 3.2	0.034	1.3	−0.4; 2.9	0.135*
Population between 16 and 20 years density^¥^	3.0	1.5; 4.5	<0.001	1.5	−0.1; 3.1	0.060*
Average family income per capita^¥^	−3.6	−5.1; −2.1	<0.001	−1.8	−3.4; −0.1	0.040*
Connectivity between four or more streets^¥^	0.3	−1.2; 1.9	0.669	0.8	−0.6; 2.2	0.254
Street lighting proportion^¥^	0.6	−0.9; 2.2	0.397	2.2	0.5; 3.9	0.013*
Paved streets proportion^¥^	−2.3	−3.8; −0.8	0.003	−2.1	−3.6; −0.7	0.004*
Sidewalks proportion^¥^	0.2	−1.3; 1.7	0.795	0.2	--1.2; 1.6	0.749
Trees existence proportion^¥^	0.4	−1.1; 1.9	0.607	0.6	−0.8; 2.0	0.424
Open sewage proportion^¥^	2.1	0.6; 3.7	0.006	0.4	−1.0; 1.9	0.546
Spread garbage proportion^¥^	1.5	−0.1; 3.0	0.059	0.9	−0.6; 2.3	0.234
Public open spaces	−0.7	−1.2; −0.2	0.008	−0.5	−1.0; 0.0	0.053*
Public open spaces with at least on physical activity attribute	−0.4	−1.1; 0.4	0.320	−0.4	−1.1; 0.2	0.190
Public open spaces with at least on physical activity attribute of regular quality	−0.9	−1.8; −0.1	0.036	−0.6	−1.4; 0.2	0.133
Public open spaces with at least on good physical activity attribute	−1.1	−2.2; 0.0	0.056	−0.7	−1.7; 0.4	0.191
Walking paths/trails	−1.0	−1.8; −0.1	0.026	−0.6	−1.3; 0.2	0.170
Football pitches	0.7	0.0; 1.4	0.058	0.0	−0.6; 0.7	0.968
Cycle paths/lanes	−3.2	−5.9; 0.6	0.017	−1.1	−3.6; 1.3	0.374
Gym	−0.7	−1.1; −0.3	0.001	−0.2	−0.7; 0.1	0.185
Beachfront	2.3	−11.4; 15.9	0.745	1.33	−11.3; 14.0	0.836
Total attributes to physical activity practice	−0.4	−0.7; −0.1	0.005	−0.2	−0.5; 0.1	0.142
Lower distance for any physical activity attribute^¥^	0.3	−1.2; 1.9	0.669	−0.8	−0.6; 2.2	0.254

^¥^Standardized variables (transformed into Z-scores)

^#^Wald test, Multiple Linear Regression

**Adjusted for sex, time living at that address and socioeconomic status

*Additional adjustment – Adjusted for sex, time living at that address and socioeconomic status and other environmental variables associated (*p* < 0.05) in the first adjusted model

Public street lighting and the existence of trees in the area were significantly associated with increased self-reported walking after adjustment for sex, time living in that address and SES. However, both variables were no longer associated after being included simultaneously in the final adjusted model (Table 3).

**Table 3:** Crude and Adjusted associations between built environmental variables and leisure walking practice for leisure (none vs. some; *N* = 3379)

Variables	Crude analyses			Adjusted analyses**		
	OR	95%CI	p^#^	OR	95%CI	p^#^
Population density^¥^	1.02	0.95; 1.10	0.525	1.02	0.95; 1.09	0.613
Population between 16 and 20 years density^¥^	0.99	0.92; 1.06	0.779	0.99	0.92; 1.07	0.868
Average family income per capita^¥^	1.07	1.00; 1.15	0.050	1.04	0.97; 1.13	0.282
Street lighting proportion^¥^	1.10	1.02; 1.19	0.013	1.00	0.87; 1.14	0.967*
Paved streets proportion^¥^	1.01	0.94; 1.08	0.842	1.00	0.93; 1.08	0.963
Sidewalks proportion^¥^	1.01	0.94; 1.09	0.775	1.01	0.94; 1.08	0.812
Trees existence proportion^¥^	1.11	1.03; 1.20	0.006	1.11	0.97; 1.26	0.132*
Open sewage proportion^¥^	0.96	0.89; 1.03	0.227	0.98	0.90; 1.05	0.524
Spread garbage proportion^¥^	0.99	0.93; 1.07	0.881	1.00	0.93; 1.07	0.971
Public open spaces	1.00	0.97; 1.02	0.899	0.99	0.97; 1.02	0.593
Walking paths/trails	1.00	0.96; 1.04	0.854	1.00	0.96; 1.04	0.842
Gym	0.99	0.97; 1.01	0.405	0.98	0.96; 1.00	0.125
Beachfront	1.60	0.85; 2.92	0.145	1.62	0.87; 3.01	0.127
Lower distance for any physical activity attribute^¥^	0.98	0.91; 1.05	0.508	0.99	0.92; 1.07	0.793

^¥^Standardized variables (transformed into Z-scores)

^#^Wald test, Multiple Logistic Regression

**Adjusted for sex, time living at that address and socioeconomic status

*Additional adjustment – Adjusted for sex, time living at that address and socioeconomic status and other environmental variables associated (*p* < 0.05) in the first adjusted model

The buffer’s mean family income, higher open sewage proportion, number of public open spaces, gyms and beachfront existence were significant environmental predictors of self-reported leisure time MVPA in crude analyses. After adjustment for sex, time living in that address and SES, beachfront existence, 16–20 years’ population density and spread garbage proportion were included in the final model, where beachfront existence increased more than three times the odds of leisure-time MVPA practice (OR = 3.31; 95%CI: 1.37; 8.02, *p* = 0.008). The other variables were no longer statistically significant (Table 4).

**Table 4:** Crude and adjusted associations between built environmental variables and leisure moderate to vigorous physical activity practice (none vs. some; *N* = 3379)

Variables	Crude analyses			Adjusted analyses**			
	OR	95%CI	p^#^	OR	95%CI	p^#^	
Population density^¥^	0.96	0.89; 1.02	0.202	0.96	0.89; 1.03	0.268	
Population between 16 and 20 years density^¥^	0.91	0.85. 0.97	0.006	0.95	0.88; 1.03	0.210*	
Average family income per capita^¥^	1.14	1.06; 1.22	<0.001	1.06	0.98; 1.15	0.168	
Street lighting proportion^¥^	1.06	0.99; 1.14	0.071	1.04	0.96; 1.12	0.318	
Paved streets proportion^¥^	0.99	0.92; 1.06	0.719	0.98	0.91; 1.02	0.576	
Sidewalks proportion^¥^	0.97	0.91; 1.04	0.395	0.97	0.90; 1.05	0.473	
Trees existence proportion^¥^	1.04	0.97; 1.11	0.321	1.02	0.94; 1.10	0.618	
Open sewage proportion^¥^	0.91	0.84; 0.97	0.006	0.98	0.90; 1.06	0.562	
Spread garbage proportion^¥^	1.05	0.98; 1.12	0.145	1.08	0.99; 1.17	0.050*	
Public open spaces	1.03	1.01; 1.06	0.009	1.01	0.99; 1.04	0.395	
Public open spaces with at least on physical activity attribute	1.01	0.97; 1.04	0.753	0.99	0.95; 1.03	0.526	
Public open spaces with at least on physical activity attribute of regular quality	1.04	1.00; 1.09	0.051	1.00	0.96; 1.05	0.842	
Public open spaces with at least on good physical activity attribute	1.04	0.99; 1.09	0.173	0.99	0.94; 1.05	0.789	
Walking paths/trails	1.03	0.99; 1.07	0.148	1.00	0.95; 1.04	0.837	
Football pitches	0.98	0.95; 1.01	0.155	0.99	0.95; 1.02	0.481	
Cycle paths/lanes	1.10	0.98; 1.25	0.114	0.98	0.86; 1.13	0.829	
Gym	1.03	1.01; 1.05	0.002	1.02	0.99; 1.04	0.173	
Beachfront	3.38	1.50; 7.63	0.003	3.31	1.37; 8.02	0.008*	
Total attributes to physical activity practice	1.02	1.01; 1.03	0.006	1.01	0.99; 1.02	0.462	
Lower distance for any physical activity attribute^¥^	0.98	0.92; 1.05	0.616		0.98	0.91; 1.06	0.586

^¥^Standardized variables (transformed into Z-scores)

^#^Wald test, Multiple Logistic Regression

**Adjusted for sex, time living at that address and socioeconomic status

*Additional adjustment – Adjusted for sex, time living at that address and socioeconomic status and other environmental variables associated (*p* < 0.05) in the first adjusted model

While gender interaction in the association between built environment and physical activity was not identified, we found a built environment by SES interaction for the associations with commuting physical activity. Table 5 presents adjusted analyses stratified by SES tertiles. In the final model including environmental variables, street lighting proportion (OR = 1.22; 95%CI: 1.01; 1.47, *p* = 0.035) and cycle path/lanes (OR = 1.77 95%CI: 1.05; 2.96, *p* = 0.031) were positively associated with commuting physical activity. Furthermore, among those from the top SES tertile, commuting physical activity was inversely associated with cycle and walking paths. No associations were found in any adjusted model among those participants from the bottom SES tertile (poorest).

**Table 5:** Adjusted** associations between built environmental variables and commuting physical activity stratified by socioeconomic status (SES) (none vs. some; *N* = 3379)

Variables	SES 1° tertile			SES 2° tertile			SES 3° tertile		
	OR	95%CI	p^#^	OR	95%CI	p^#^	OR	95%CI	p^#^
Population density^¥^	1.16	0.94; 1.44	0.163	1.13	0.95; 1.36	0.175	1.11	0.93; 1.31	0.248
Population between 16 and 20 years density^¥^	1.22	0.99; 1.50	0.060	1.13	0.94; 1.36	0.194	1.14	0.95; 1.37	0.160
Average family income per capita^¥^	0.95	0.75; 1.21	0.697	1.03	0.80; 1.31	0.838*	1.09	0.92; 1.28	0.315
Street lighting proportion^¥^	1.06	0.87; 1.28	0.587	1.22	1.01; 1.47	0.035*	0.95	0.80; 1.13	0.583
Paved streets proportion^¥^	0.82	0.67; 1.00	0.050	0.95	0.80; 1.13	0.553	1.01	0.85; 1.20	0.892
Sidewalks proportion^¥^	0.89	0.72; 1.10	0.290	1.01	0.85; 1.22	0.878	1.01	0.86; 1.20	0.874
Trees existence proportion^¥^	1.03	0.84; 1.26	0.784	1.19	0.99; 1.44	0.061*	1.02	0.83; 1.25	0.866*
Open sewage proportion^¥^	1.12	0.91; 1.37	0.277	0.95	0.77; 1.15	0.591	0.86	0.73; 1.02	0.075*
Spread garbage proportion^¥^	0.85	0.70; 1.03	0.107	1.21	0.99; 1.49	0.063	0.88	0.75; 1.04	0.130
Connectivity between four or more streets^¥^	1.30	0.99; 1.70	0.059	1.14	0.90; 1.43	0.269	1.13	0.95; 1.34	0.156*
Public open spaces	1.04	0.95; 1.14	0.417	0.98	0.92; 1;04	0.477	1.00	0.95; 1.05	0.918
Walking paths/trails	1.01	0.87; 1.17	0.943	1.07	0.95; 1.21	0.281	0.87	0.80; 0.95	0.001*
Cycle paths/lanes	0.95	0.59; 1.52	0.825	1.77	1.05; 2.96	0.031*	0.65	0.50; 0.83	0.001*
Beachfront	-	-	-	0.46	0.13; 1.70	0.246	1.41	0.32; 6.19	0.646
Lower distance for any physical activity attribute^¥^	0.89	0.78; 1.01	0.061	0.98	0.79; 1.22	0.858	0.96	0.82; 1.14	0.678

^¥^Standardized variables (transformed into Z-scores)

^#^Wald test, Multiple Logistic Regression

**Adjusted for sex and time living at that address

*Additional adjustment – Adjusted for sex, time living at that address and socioeconomic status and other environmental variables associated (*p* < 0.05) in the first adjusted model

## Discussion

The present study evaluated associations between objectively measured built environment and total, domain- and type-specific physical activity. Some significant associations were observed in the expected direction, even after adjustment for SES and other significant environmental variables. Street lighting was significantly associated with objectively measured MVPA in the whole sample and with commuting physical activity among participants from the intermediate SES tertile. The proportion of paved streets and buffer’s average family income were inversely associated with objectively-measured MVPA. Living close to the beachfront was a significant correlate of leisure-time MVPA. Cycle path/lane was a positive correlate to commuting physical activity among participants from the intermediate SES. In contrast to our expectations, among those from the wealthiest SES tertile, cycle paths and walking paths/trails were inverse correlates of commuting physical activity, even after adjustments for other environmental variables.

Specific characteristics of the city may also help to contextualize and to interpret these results; Pelotas is located near the coast in the extreme south of Brazil and is surrounded by a huge lagoon. The beachfront seems to be the public place most used by general population and the public green areas are scarce. Climate is also peculiar, characterized by higher humidity and regular precipitation in all seasons. There is no policy promoting physical activity, except the building of a few cycle paths in the 3 years preceding the study, mostly in the city centre or richer areas of the city.

Studies examining the associations between the built environment and youth physical activity levels have increased during the past two decades, but most associations are still inconclusive, particularly from low and middle-income countries, where few studies are available [3]. In the most comprehensive review so far, Bauman and co-workers [3] highlighted that neighbourhood design, recreation facilities, and transportation systems were consistently related to physical activity among youth. It is important to note that associations between built environment attributes and physical activity are influenced by the measurement techniques employed in each study. For example, objective measures of the built environment might provide more accurate data, and therefore more consistent associations, as compared to subjective techniques [18].

Studies among adolescents using objective measures of the environment found that land-use mix and residential density were the most frequent significant correlates of transport-related physical activity [18]. Furthermore, access to parks, recreation facilities and street connectivity were associated in the expected direction in 47%, 43% and 48% of the studies, respectively [18]. We observed similar associations regarding urban form, indicated by the associations between physical activity of population density and street connectivity. However, after adjusting for other environmental variables, these two environmental characteristics were no longer significant. In terms of parks and recreation facilities, our study added evidence on the absence of association between leisure-time physical activities and public open spaces. In addition, a feature possibly related to safety, public street lighting was positively associated with leisure-time walking and, specifically among those from the intermediate SES tertile, with commuting physical activity. The proportion of paved streets within buffers, which might be considered an indicator of safety from traffic, was inversely associated with total MVPA in our sample.

Built environment associations with commuting physical activity were modified by SES. Stratified analyses highlighted the lack of associations between environmental characteristics and commuting physical activity among those participants from the poorest SES tertile, stronger associations among those from the intermediate tertile, and some unexpected associations among those from wealthiest SES group (commuting physical activity was inversely associated with cycle and walking paths). Commuting physical activity, mainly in low and middle-income settings, might be more related to a need (e.g. lack of financial or infrastructure support) than to an individual choice. It is usually more frequent among low SES groups, despite the lower availability/quality of built environment characteristics compared to their counterparts. Moreover, the intermediate SES tertile may represent a group exposed to more environmental variability, justifying the findings of the present analyses.

Studies assessing built environment correlates of PA among Brazilian adolescents appear non-existent. Some studies among adult populations are available [19–21]. In Curitiba, a Brazilian city characterized by a planned urbanization and large number of public open spaces, leisure-time walking and MVPA were related to higher income and higher number of gym clubs in the buffer. The Curitiba study also found increased odds for walking for those living near recreational centres [19]. Commuting walking and leisure-time MVPA were also positively associated with the ‘walkability index’ in Curitiba [20].

Most of the associations, particularly those related to public open spaces, were non-significant. Possible explanations are the low variability of physical activity attributes and unequal distribution of public open spaces around the city. A larger number of public open spaces was found in census tracts with higher average family income. Exactly among these families, people usually have a wider option of choice for their leisure-time physical activities. In addition to public open spaces showing general poor quality and unsuitability for physical activity practice, other possible aspects such as social insecurity and fear of crime may explain this lack of association.

The present study has some limitations. The environment surrounding participants’ work/school/university was not assessed and people might practice physical activity in these areas. Further, private places for indoor football practice were not evaluated in our environmental analysis, which may limit the possibility to observe associations particularly among males, as this is a common activity for them. Finally, the cross-sectional nature of the data impedes us from evaluating temporality of the observed associations.

Built environment characteristics may be assessed also based on participants’ perceptions about the place where they live. Objective and subjective assessments provide different and complementary information. While subjectivity provides participant’s feeling about the existent built environment, this perception may be influenced by previous behaviours and previous experiences [22]. Objective measures might be considered more accurate to describe a wide number of built environment variables, such as distance to certain places and existence of green areas around individuals’ households. Moreover, objective measures in this field enable an easier translation between evaluation and future actions, making studies more relevant for researchers and practitioners [8]. Nevertheless, most of the available geographical information was not collected for the purpose of studying its association with physical activity. The evaluation of environmental characteristics surrounding participants’ households, for example, are based on secondary data provided by the National Demographic Census, and, despite its standardized procedures of data collection, the quality of information could not be guaranteed. GIS data also tend to collapse information collected in different periods [8], and information about the quality of the attributes is rarely available. For the current analyses, most environmental information was collected within a short time-frame as the physical activity data collection, except the information on green areas provided by Pelotas’ street network, which were collected with urban planning purposes. In order to minimize this limitation, we conducted a study to update this information.

Despite the large number of statistical tests performed, increasing the likelihood of random significant findings (type 1 error), an additional strength of the present study was to evaluate a wide range of built environment variables and their influence on overall, domain and type-specific physical activities [8, 18]. Theoretical models assume a higher specificity in environmental influences on different physical activity practices and their context [18, 23].

## Conclusion

Improvements in the built environment, especially in characteristics such as public open spaces, cycle and walking paths, are essential to promote physical activity worldwide. The debate on whether investments should focus on the individual or environmental levels typically concludes that both investments are needed. Behaviour modification is a product of individual choices and the surrounding environment. Thus, particularly in low and middle-income countries, investments in improving access to physical activity are needed. Furthermore, environmental-based interventions tend to have a wide coverage and might influence large groups or entire populations [24]. In our study, some environmental attributes such as beachfront, street lighting, paved streets and cycle paths/lanes were shown to be associated with physical activity patterns. Infrastructure changes which improve these environmental factors may therefore influence physical activity levels of southern Brazilian citizens.

## Abbreviations

GIS: Geographic information system
MVPA: Moderate to vigorous physical activity
PARA: Physical activity resource assessment
SES: Socioeconomic status
